# Resequencing DCDC5 in the Flanking Region of an LD-SNP Derived from a Kidney-Yang Deficiency Syndrome Family

**DOI:** 10.1155/2011/215653

**Published:** 2011-05-05

**Authors:** Li Ping Zhou, Wei Wei Liu, Tian E. Zhang, Wei Hong Li, Ling Ling Tan, Wan Zhen Li, Yu Hua Qin, Hong Ya Yang, Azure Duan, Mi Qu Wang, Wei Jun Ding

**Affiliations:** Department of Fundamental Medicine, Chengdu University of Traditional Chinese Medicine, Chengdu 610075, China

## Abstract

*Objective*. To explore the genetic traits of Kidney-yang deficiency syndrome (KDS). *Design*. Twelve KDS subjects and three spouses from a typical KDS family were recruited. Their genomic DNA samples were genotyped by Affymetrix 100K single-nucleotide polymorphism (SNP) arrays. The linkage disequilibrium (LD) SNPs were generated using GeneChip DNA analysis software (GDAS, Affymetrix). Genes located within 100 bp of the flanks of LD SNPs were mined via GeneView. 29 exons of the doublecortin domain containing 5 (DCDC5), a representative gene within the flank of an LD SNP, were resequenced. *Results*. Five LD SNPs display midrange linkage with KDS. Two genes with established functions, DCDC5 and Leucyl-tRNA synthetase, were mined in the flanks of LD SNPs. Resequencing of DCDC5 revealed a nonsynonymous variation, in which 3764T/A was replaced by C/G. Accordingly, the Ser^1172^ was substituted by Pro^1172^. The S1172P substitution effect was evaluated as “possibly damaging” by PolyPhen. *Conclusion*. We have identified a genomic variation of DCDC5 based on the LD SNPs derived from a KDS family. DCDC5 and other genes surrounding these SNPs display some relationships with key symptoms of KDS.

## 1. Introduction

The genetic features of kidney-yang deficiency syndrome (KDS) still remain obscure. However, there have been few investigations that have focused on genetic alterations that might correlate with this disease. Wang et al. [1] suggested that the cause and progression of KDS involves not only environmental factors but genetic elements as well. Pan et al. [2] analyzed the transcriptomic patterns of KDS subjects utilizing microarrays. The KDS transcriptome module was further analyzed by Yang et al. [3] and Wu et al. [4]. However, these studies did not identify any genetic alterations associated with this disease. Thus it was the purpose of this study to identify possible genetic alterations that could contribute to the development of KDS.

Re-sequencing is a promising method that is particularly valuable for the genetic studies of complex diseases. For example, Dapprich et al. [5] described a simple and automated method of targeting specific sequences of genomic DNA linked to SNPs of interest and associated with certain complex traits or drug responses. Smyth et al. [6] tested the shared and distinct non-HLA loci between two inflammatory disorders, type 1 diabetes, and celiac disease, indicating that a genetic susceptibility to both diseases shares common alleles. Wermter et al. [7] employed resequencing and relevant methods to analyze the mutations of melanin-concentrating hormone receptor 1 gene and reported that several alleles and/or haplotypes might be involved in juvenile-onset obesity. Indeed, nucleotide sequencing, originally described by Sanger and colleagues, is still the gold standard for the identification of genetic mutations and polymorphisms.

KDS and other TCM syndromes share many features with complex diseases. TCM, by its sheer nature, is based on the analysis of the simultaneous occurrence of pathological processes on a macroscopic level and thus provides a holistic approach complementary to the trend toward disaggregation of complex phenotypes as is practiced in conventional medicine [8]. For example, a typical complex disease, diabetes, has been traditionally named by TCM as thirst-and-emaciated syndrome, exactly manifesting the major symptoms of diabetes. Hence, we propose a hypothesis that TCM syndromes, just like hypertension, diabetes, and other complex diseases defined by Western medicine, possess intricate genetic traits involving DNA structure variations, such as SNP alleles, SNP linkage disequilibrium (LD), and gene mutations.

The purpose of this paper is to explore the DNA structure variations of KDS by a resequencing approach. Based on the importance of the resequencing method upon the genetic traits of complex diseases, and the shared features between complex diseases and TCM syndromes, we adopted this method to investigate genomic variations of KDS. We firstly employed a classic method universally applied in human genetics, pedigree approach, to report that KDS patients undergo overall attenuated functions in the mass-energy-information-carrying network [9]. Second, we utilized a genome-wide method, SNP array, to retrieve the LD SNPs within the KDS family [8]. In the present paper, we employed resequencing to identify the defined DNA mutations of genes located within 100 bp of the flanks of LD SNPs, and we report the results of LD SNPs and sequencing polymorphisms of doublecortin domain containing 5 (DCDC5), an important gene that may play an essential role in the pathology of KDS.

## 2. Materials and Methods

### 2.1. Collection of Genomic DNA Samples from a KDS Family

This work was approved by the Ethics Board, Chengdu University of TCM, and all participants signed the informed consent form before examination [8]. The process of collecting KDS pedigree was described previously [8, 9]. Briefly, we used a 40-item scoring table of KDS as a criteria to recruit KDS subjects in Chengdu, southwest China, and defined participants as KDS subjects (total score ≥12 points) or healthy individuals (total score ≤5 points). In order to improve the validity and reproducibility of the TCM diagnosis, every participant was diagnosed by five independent TCM physicians, and the examinations were repeated for three consecutive years (from 2003 through 2005), all on the first Saturday of December [8, 9].

Three mL of blood sample was collected from each subject. The genomic DNA was isolated by conventional phenol/chloroform extraction, washed with 95% ethanol, re-suspended in TE buffer, and stored at −80°C until further use.

### 2.2. Genotyping, LD Estimating, and Mining the Genes around LD SNPs

The detailed description of these procedures has been reported previously [8, 9]. Briefly, DNA samples were genotyped with the Affymetrix GeneChip Mapping 100K kit, at a commercial laboratory (National Engineering Center for Biochip at Shanghai, China). Genotype data of all the subjects were generated using a GeneChip DNA analysis Software (GDAS, Affymetrix). The pedigree information, allele frequencies, and map position of the SNPs were combined with the genotype data. To determine the LD SNPs from this KDS family, we performed a simulation study using Agilent Varia software [10, 11]. The following steps were automatically executed in the computer workstation of the laboratory. (a) likelihoods, *L* (*θ*), are calculated for each possible *θ* value ranging from 0 to 0.5, in increments of 0.02, that is, 0, 0.02, 0.04,…, 0.46, 0.48, 0.5. (b) Choose *θ* which maximizes the likelihood ratio, *L*(*θ*)/*L*(1/2), of the occurrence of linkage to no linkage. (c) Calculate the lod adds (LOD) score for this *θ.*

Genes located within 100 bp of the flanks of LD SNPs were mined via an authoritative web tool, GeneView (http://www.ncbi.nlm.nih.gov/SNP/). Their biological functions were then annotated by employing FatiGO (http://fatigo.bioinfo.ochoa.fib/es).

### 2.3. Re-Sequencing of Doublecortin Domain Containing 5 (DCDC5)

All of the 29 exons of DCDC5 were covered by 29 pairs of PCR primers (Table 1). These primers were designed based on the DCDC5 version issued on 26 June, 2007 (NM_198462.2). A website primer design platform, Primer3 (http://www.genome.wi.mit.edu/cgi-bin/primer/primer3.cgi), was employed under the default primer selection conditions. All samples, including twelve KDS patients and three spouses from the KDS pedigree were resequenced and compared for variation discovery in DCDC5. A detailed laboratory protocol can be found on the SeattleSNPs website (http://pga.gs.washington.edu/). PCR products were sequenced using each pair of above PCR primers and BigDye Terminator Cycle Sequencing v2.0 kit (Applied Biosystems, Applera, USA). Sequencing reactions were electrophoresed on an ABI 377 automated sequencers. Finally, the substitution effect was evaluated by a free software, PolyPhen (http://genetics.bwh.harvard.edu/pph/).

## 3. Results

### 3.1. General Conditions of a Representative KDS Family

**Figure 1:**
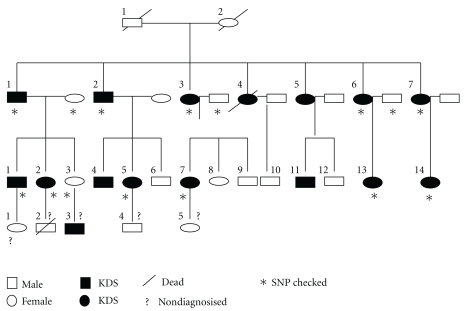
Pedigree tree of the collected KDS family. This family comprise four generations and 35 members that display an autosomal dominant model of inheritance. The KDS subjects were evaluated by a 40-item scoring table based on TCM criteria system of KDS promulgated by the Health Department of China (GB/T15657-1995). Dark color indicates KDS. The mark “?” indicates those were not evaluated due to death, nonaccessibility, or babies who were too young to be easily checked. The mark “∗” indicates those genome SNP were checked by SNP chip. KDS: kidney-yang deficiency syndrome. TCM: traditional chinese medicine. SNP: single nucleotide polymorphism.

A typical KDS pedigree, including 17 KDS patients distributed in four generations who live in the area surrounding Chengdu, China, was selected as the subject for the present study. Their KDS symptoms were confirmed by repeated TCM diagnosis and clinic biochemistry assays [8]. Twelve available KDS subjects (Figure 1) were collected for genetic analysis. Simultaneously, three healthy spouses, whose life environment and ages were best matched with those of the KDS subjects, were recruited as non-KDS controls.

### 3.2. Two Genes with Established Functions Were Mined in the Flanks of LD SNPs

Five SNPs obtained from the KDS family were identified as LD SNPs. The sequences of alleles of LD SNPs identified from the KDS family were displayed in Table 2. It has been defined that *θ* ≤ 0.10 indicates tight linkage; while 0.10 < *θ* < 0.20, midrange linkage and *θ* ≥ 0.20, loose linkage [10]. Results displayed that before their *θ* values achieved 0.2, all LOD values of these SNPs were greater than 1.0. Hence, these LD SNPs demonstrated midrange linkage with KDS.

**Figure 2:**
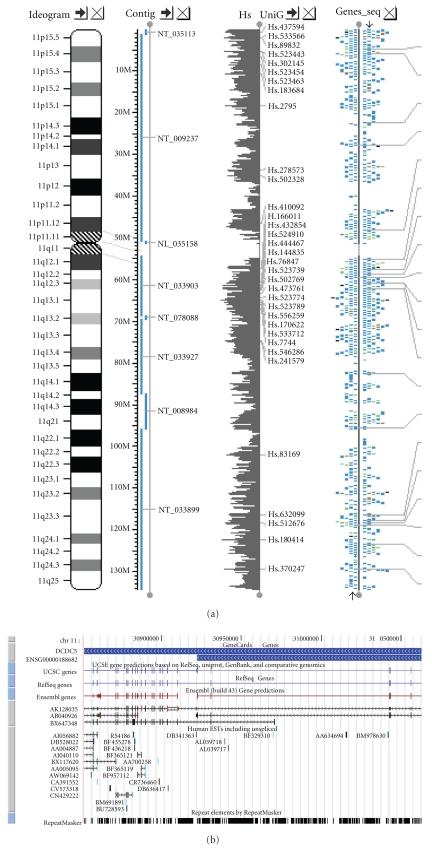
Chromatograms of the LD SNPs. The upper part illustrates chromosome 11p14.1-p13 and flanks with respect to the gene DCDC5. The lower part is the result of linkage analysis corresponding to the assay of SNP arrays based on a KDS family.

We then examined the genes within 100 base pairs of flanks of these LD SNPs (Figure 2), and observed two genes with established functions. One gene, doublecortin domain containing 5 (DCDC5, NM_198462), is referred to rs514207. DCDC5 is located in chromosome 11p14.1-p13. The established functions of DCDC5 are sugar binding, interacting selectively with mono-, di-, or trisaccharide carbohydrate, and involving certain intracellular signaling cascades [12–17]. Thus, DCDC5 might be one of the key factors that monitor saccharides and energy metabolism. Another gene, encoding Leucyl-tRNA synthetase (LARS), is located near rs1054020. It is also a pleiotropic gene that involved in cysteine-tRNA ligase activity, tRNA aminoacylation, ATP binding, protein biosynthesis, and other biological processes [18, 19].

### 3.3. SER/PRO Alteration Was Observed in Exon 27 of DCDC5

**Figure 3:**
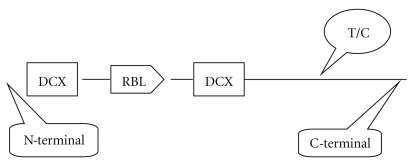
Schematic representation of the Ser/Pro substitution occurred in DCDC5. Totally that there are 1258 residues of amino acids. The first and second DCX domains are located at 4–94 and 491–570, respectively. The Rincin-B-Lectin (RBL) domain is located at 238–376. DCDC5: doublecortin domain containing 5. DCX: doublecortin.

Results of resequencing of DCDC5 show several variations and/or mutations (Table 3). It is worth noting that in eight of the twelve KDS participants, the 3764T/A was replaced by C/G. As a nonsynonymous variation, the wild-type Ser^1172^ of DCDC5 was substituted by Pro^1172^ in the vicinity of C-terminal (Figure 3). This novel variation occurred within exon 27 of DCDC5. This mutation has not been previously reported and does not show any relationship with the established variants of DCDC5 [12–17]. Results of the tolerable or deleterious probability for S1172P substitution were +1.040 for score 1, and −0.462 for score 2, which could be marked as “possibly damaging” by PolyPhen. 

## 4. Discussion

A resequencing approach, based on the results of LD SNPs, is a promising strategy for exploring the genetic traits of complex diseases. Most functionally important SNPs are located within genes and other critical gene regulatory regions, such as the promoters, 5′ and 3′ untranslated regions, and predicted splice sites [20–23]. Therefore, SNPs can alter gene  expression and phenotypes through a variety of mechanisms. Thus far, only a few SNPs have been identified as LD SNPs representing strong candidate biomarkers for certain diseases [8, 10, 22, 23]. However, given the extreme complexity of genomic structure, resequencing and relevant methods that can identify the specific variants in a sequence of interest compared with a known genomic sequence is a monumental task [22]. Hence, combined analysis of LD SNPs and resequencing can focus on the targets and identify the defined variations, especially in the exploration of genetic characteristics of complex diseases. Results of this present work also reveal that sequence analysis associated with LD SNP analysis is likely to be a useful tool to substantially increase our understanding of the genetic basis of KDS.

Results of resequencing reveal a novel variation occurring in the vicinity of the C-terminal of DCDC5. Nagase et al. [13] cloned DCDC5 from a fetal brain cDNA library. This gene is located in chromosome 11p14.1-p13 and is weakly expressed in male reproductive system, testies and eyes. Thus far, four transcripts, several amino acid variants and about 100 SNPs of DCDC5 have been documented [12]. In the present work, a novel genomic variant of DCDC5 was identified in family members suffering from KDS. Accordingly, within the NCBI reference sequence NP_940864.2, the Ser^1172^ was replaced by Pro^1172^. This variant has not been found in any established domains of DCDC5, nor is it located in any active enzyme site (e.g., enzyme site for serine/threonine protein kinase). This substitution could possibly damage the structure and/or function of DCDC5. Thus, functional consequences of this causative variant in DCDC5 associated with susceptibility to KDS remains to be confirmed. Notwithstanding a functional change in the protein, our findings do document a new variant associated with KDS, characterized by a novel SNP within the genomic region of DCDC5. To our knowledge, there have been no previous studies reporting genomic variations associated with TCM syndrome.

As a relatively new studied gene, DCDC5 is of importance in saccharide binding, energy metabolism and diverse signal transduction pathways. Recent reports [12, 13] revealed that DCDC5 located in chromosome 11, one of the most gene- and disease-enrich chromosomes in the human genome. Human DCDC5 contains two DCX domains separated by a ricin-like domain (RICIN_B_LECTIN) (Figure 2). DCX domains serve as protein-interaction platforms and are likely to mediate signal transduction pathways. Mutations in DCX genes result in abnormal neuronal migration, epilepsy, mental retardation, inherited blindness, and dyslectic reading disabilities [12]. However, the quest for how DCX domain-containing proteins exert their multiple functions is still unresolved [15]. In contrast, RICIN_B_LECTIN domains interact selectively with mono-, di-, or trisaccharide carbohydrates [16] and involve signaling cascades such as the regulation of cytoskeleton [17, 18]. Therefore, DCDC5 is an important and pleiotropic gene involving diverse functions such as saccharides binding, energy metabolism, cell cycles, and intracellular signaling cascades.

DCDC5 is likely to be involved in the physiological functions and pathological process of KDS. From the viewpoint of TCM, the major biological functions of DCDC5 could be included into the TCM concept of the kidney. TCM kidney, as a critical monitor of metabolic, immune, and reproductive systems, works closely with the neural-endocrine-immune network [8, 9, 24]. Consequently, many vital functions and activities of KDS patients might be globally attenuated or disrupted. The major symptoms of KDS, such as dislike of cold, persistent cold extremities, and favor of warmth are mostly due to the insufficient energy metabolism, thus resulting in an abnormal low level of energy output [24–27]. Apparent overlap in major physiological functions and pathology between DCDC5 and KDS suggests certain relationships that still need to be determined. In other words, DCDC5 is an important but less studied gene that is worthy of consideration for further analysis by scientists investigating the pathology of KDS.

The main limitation of this work is that we only examined exons of DCDC5. Variations located within other critical sites of DCDC5, such as the introns, promoters, 5′ and 3′ untranslated regions, and predicted splice sites, have not been resequenced. Further, we only checked twelve KDS subjects from a typical KDS family. Therefore, analysis of other KDS families and individuals with KDS are necessary. 

In conclusion, we report in this paper a genomic variation of DCDC5, which could possibly damage this protein, based on the analysis of LD SNPs from a KDS family. DCDC5 and other genes located in the flanks of these SNPs display relationships with key symptoms of KDS. However, further work is required to study their function in order to establish the detailed relationships of DCDC5 to KDS. The merits of linkage analysis combined by resequencing may warrant further exploration of the genetic features of TCM syndromes. Regardless, our current data provides new and relevant information on gene associations that may correlate with the development and progression of KDS.

## Figures and Tables

**Table 1:** Primers designed for the resequencing of DCDC5. A website primer design platform, Primer3, was employed under the default primer selection conditions to design these primers based on the genbank accession number NM_198462.2. All samples, including twelve KDS patients and three spouses from the KDS family were resequenced and electrophoresed on an ABI 377 automated sequencers.

Exon	Forward primer	Reward primer
1	taaccccttcaggtgagcag	caatcagtcacctggccttt
2	ggagaagcaggagcctttct	gtatcaaaaccagggccaag
3	aacattgccgtcatttcaca	actgccttgcaaaaacacct
4	ccagcaatagtgctatacaataggaa	atggccccatatggttttct
5	ccccaatgactgctggtact	ccagctacctgaaaggctga
6	caggtggggagagagagaga	aataccaacggcagagatgg
7	ggctcgctgactgctaagtt	tgttgaaggccaacatttca
8	gccaggctatctggaaaatg	gggaattgctgggttgtcta
9	tcctctctttccagttggatg	acgtggccaaaagaaaagaa
10	ttggccacgtacaaaggact	ctacgcttgaaagccaaagg
11	tttgccttaatgctttcctga	gaccacacaactgggcctta
12	ttccaagttcattcggttct	aatatgggagcccttttgct
13	gccacaatttggagaggaaa	tccagcctgggtatcagagt
14	tgttgcctttatcagcagtttg	agcaagatgcagaggtgaaa
15	ggactgggacttccactgat	ttcacaaacttggcatgagc
16	ccaccaacatcctgtggaat	caggaggcaaaggtggataa
17	tggagctctcaccaacagaa	ccaggagatcagagggaaca
18	cccttcatcctgcagtcttc	cttgccattgggtttgattt
19	tgtcactgtgtggtgggatta	tttcactcaaaaatcagcattca
20	tcattggtgtcgaattgtcg	caatggggaaaaggaaatca
21	gccagaaacacaagccagat	aatctggccagggacattta
22	cagctggattggactactgttg	ttgacactcttcgggtcaca
23	gattttggcattgcctgttt	ggcaaatgtgcaaacatgag
24	tgccttaagccctgtcatct	ttgcccaggtatcctcctaa
25	gctcctgtgtggaacgattt	gctgggcctttttctcttct
26	aagcaaggggaatttaccaga	tctgctggacaagttgcctat
27	acatttgcatgcccttcttc	gcgattgaattctccagagc
28	ggatttgtgaggggtcttca	caaggctgcagtaagctgtg
29	gtgtgtgcgtgtgttcatgt	tcaaaataggcccattgaaaa

**Table 2:** Five SNPs of linkage disequilibrium derived from a KDS family. A conventional approach, lod adds Score method (LODs), was employed to calculate the levels of recombination condition. The LOD values (or Z value) were reckoned at different *θ* values (*θ* = 0 ~ 0.5). The criteria standard of linkage are, *Z* > 1: support linkage, *Z* > 3: linkage. Accordingly, *θ* ≤ 0.10 indicates tight linkage, 0.10 < *θ* < 0.20, midrange linkage, *θ* ≥ 0.20, loose linkage.

SNP NO.	SNP1	SNP2	SNP3	SNP4	SNP5
RefSNP ID	rs514207	rs1054020	rs7685923	rs10515889	rs10516202
Physical position	11:−31035695	5:145505341	4:10758418	5:164479475	4:9802214
LOD (theta = 0.0)	2.018	2.105	2.141	2.007	2.03
LOD (theta = 0.05)	1.801	1.882	1.917	1.791	1.815
LOD (theta = 0.1)	1.578	1.651	1.686	1.568	1.596
LOD (theta = 0.2)	1.114	1.167	1.206	1.108	1.149
LOD (theta = 0.3)	0.641	0.669	0.71	0.642	0.691
LOD (theta = 0.4)	0.206	0.225	0.239	0.226	0.248
Referred gene	DCDC5	LARS	/	/	/

**Table 3:** Resequencing results of DCDC5 from a KDS pedigree.

Exon no.	Sample no.	SNP	mRNA	Protein
5	8	F221 A/G	626 G	VAL(gtg)-MET(atg)
11	1	F181 A/G	1267 G	ARG(agg)-ARG(aga)
11	2	F211 A/G	1267 G	ARG(agg)-ARG(aga)
11	8	F100 A/T	INTRON	
11	9	F124 A/T	INTRON	
12	1	F172 C/T	INTRON	
12	2	F172 C/T	INTRON	
12	8	F172 C/T	INTRON	
12	9	F152 C/T	INTRON	
23	1	F81 A/G	INTRON	
23	2	F80 A/G	INTRON	
23	8	F82 A/G	INTRON	
23	9	F72 A/G	INTRON	
**27**	**1**	**F352 A/G**	**3764 T→C **	**SER(tcc)-PRO(ccc)**
**27**	**2**	**R287 A/G**	**3764 T→C**	**SER(tcc)-PRO(ccc)**
**27**	**4**	**R285 A/G**	**3764 T→C**	**SER(tcc)-PRO(ccc)**
**27**	**5**	**R285 A/G**	**3764 T→C**	**SER(tcc)-PRO(ccc)**
**27**	**6**	**R285 A/G**	**3764 T→C**	**SER(tcc)-PRO(ccc)**
**27**	**7**	**R287 A/G**	**3764 T→C**	**SER(tcc)-PRO(ccc)**
**27**	**8**	**R282 A/G**	**3764 T→C**	**SER(tcc)-PRO(ccc)**
**27**	**9**	**R289 A/G**	**3764 T→C**	**SER(tcc)-PRO(ccc)**
28	5	F188 C/A	188 A	LEU(ctg)-ARG(cgg)
